# Local effect of stereotactic body radiotherapy for primary and metastatic liver tumors in 130 Japanese patients

**DOI:** 10.1186/1748-717X-9-112

**Published:** 2014-05-10

**Authors:** Hideomi Yamashita, Hiroshi Onishi, Yasuo Matsumoto, Naoya Murakami, Yukinori Matsuo, Takuma Nomiya, Keiichi Nakagawa

**Affiliations:** 1 Department of Radiology, University of Tokyo Hospital, 7-3-1, Hongo, Bunkyo-ku, Tokyo 113-8655, Japan; 2 Department of Radiology, University of Yamanashi, Yamanashi, Japan; 3 Department of Radiology, Niigata Cancer Center Hospital, Niigata, Japan; 4 Department of Radiation Oncology, National Cancer Center Hospital, Singapore, Singapore; 5 Department of Radiation Oncology and Image-applied Therapy, Graduate School of Medicine, Kyoto University, Kyoto, Japan; 6 Department of Radiation Oncology, Yamagata University Hospital, Yamagata, Japan

**Keywords:** Hepatocellular carcinoma, Metastatic liver tumor, Stereotactic body radiotherapy, Stereotactic ablative radiotherapy

## Abstract

**Background and aims:**

Stereotactic body radiotherapy (SBRT) is a relatively new treatment for liver tumor. The outcomes of SBRT for liver tumor unfit for ablation and surgical resection were evaluated.

**Methods:**

Liver tumor patients treated with SBRT in seven Japanese institutions were studied retrospectively. Patients given SBRT for liver tumor between 2004 and 2012 were collected. Patients treated with SBRT preceded by trans-arterial chemoembolization (TACE) were eligible. Seventy-nine patients with hepatocellular carcinoma (HCC) and 51 patients with metastatic liver tumor were collected. The median biologically effective dose (BED) (α/β = 10 Gy) was 96.3 Gy for patients with HCC and 105.6 Gy with metastatic liver tumor.

**Results:**

The median follow-up time was 475.5 days in patients with HCC and 212.5 days with metastatic liver tumor. The 2-year local control rate (LCR) for HCC and metastatic liver tumor was 74.8% ± 6.3% and 64.2 ± 9.5% (*p* = 0.44). The LCR was not different between BED_10_ ≥ 100 Gy and < 100 Gy (*p* = 0.61). The LCR was significantly different between maximum tumor diameter > 30 mm vs. ≤ 30 mm (64% vs. 85%, *p* = 0.040) in all 130 patients. No grade 3 laboratory toxicities in the acute, sub-acute and chronic phases were observed.

**Conclusions:**

There was no difference in local control after SBRT in the range of median BED_10_ around 100 Gy for between HCC and metastatic liver tumor. SBRT is safe and might be an alternative method to resection and ablation.

**Summary:**

There was no difference in local control after SBRT in the range of median BED_10_ around 100 Gy for between HCC and metastatic liver tumor and SBRT is safe and might be an alternative method to resection and ablation.

## Introduction

In Japan, an infection rate of the hepatitis C is high, and there are many hepatocellular carcinoma (HCC) cases. The liver is also a common lesion of metastases from most common solid malignancies. According to clinical practice guidelines from Japan, resection, radiofrequency ablation (RFA), and liver transplantation are the available curative options for HCC [1]. Recently, stereotactic body radiotherapy (SBRT) has become a treatment option for patients with liver tumor who are not eligible for surgery, RFA, or liver transplantation. Although HCC doesn’t really have bad radiation sensitivity [2], what’s happening now is that SBRT for HCC has not been performed very much. One of the reasons is that the role of radiotherapy (RT) for liver tumors has been limited due to the risk of radiation-induced liver disease (RILD) [3]. However, technological advances have made it possible for radiation to be delivered to small liver tumors while reducing the risk of RILD [4]. Resection, RFA, or trance-catheter arterial chemoembolization (TACE) are often performed for HCC and liver metastasis in Japan. However, only 10–20% of HCC patients have a resectable disease [5]. A drawback to RFA is that some anatomic areas make the procedure difficult to perform [6]. It is only the case with a central lesion of the liver, with direct invasion into the vessels, and/or that an effect of TACE was insufficient to be introduced to SBRT. In patients with centrally located HCC with chronic hepatitis or cirrhosis, major resection is often contraindicated due to insufficient residual liver volume [7]. RFA is therefore often contraindicated for HCC in those areas, which are located in and near the hepatic portal vein or central bile duct [8] and abutting the diaphragm [6]. Additionally, the risk of neoplastic seeding along the needle track after RFA has been reported [9].

SBRT offers an alternative, non-invasive approach to the treatment of liver metastasis. The goal of SBRT is to deliver a high dose to the target, thereby providing better local tumor control, while limiting dose to surrounding healthy tissue, thereby potentially decreasing complication rates. Early applications of SBRT to liver metastases have been promising [10-20]. While these data establish the safety of stereotactic radiation therapy for liver metastases, all SBRT treatments must be performed cautiously given the challenges of organ motion and the low radiation tolerance of the surrounding hepatic parenchyma.

Takeda *et al.*[21] reported that local control rate (LCR) after SBRT for lung metastases from colorectal cancer with a 2-year LCR of 72% was worse than that for primary lung cancer. We hypothesized that the same thing as this might apply to HCC and liver metastasis and, in other words, LCR after SBRT for liver metastases might be worse than that for HCC.

Because there was little number of cases that has performed liver SBRT in every each institution, we wanted to research results and a side effect as a whole in many institutions. The purpose of this study was to retrospectively evaluate the outcomes, mainly concerning local control, of patients treated at various dose levels in many Japanese institutions.

## Materials and methods

### Patients

This is a retrospective study to review 130 patients with primary or metastatic liver cancers treated at seven institutions extracted from the database of Japanese Radiological Society multi-institutional SBRT study group (JRS-SBRTSG). The investigation period was from May 2004 to November 2012.

The diagnosis of HCC depended mostly on imaging studies, because candidates for SBRT were unfeasible for pathological confirmation. During follow-up of patients with liver disease, nodules ≥1 cm were diagnosed as HCC based on the typical hallmarks (hyper-vascular in the arterial phase with washout in the portal, venous or delayed phases) from imaging studies, which included a combination of contrast-enhanced ultrasonography, 4-phase multi-detector computed tomography (CT), dynamic contrast-enhanced magnetic resonance imaging (MRI), and CT during hepatic arteriography and arterio-portography studies. The diagnosis was established according to a review [22] and clinical practice guidelines [23,24]. The eligibility of SBRT for HCC was a single lesion in principle.

The diagnosis of metastatic liver tumor was confirmed by diagnostic imaging including ultrasound, CT, and/or MRI. The eligibility of SBRT for metastatic liver tumor was without other lesions and in less than four.

Patient and tumor characteristics were shown in Table 1. HCC included 79 cases and the liver metastases included 51 cases. The Child-Pugh score before SBRT for HCC was 84.8% in grade A, 11.4% in grade B, and 1.3% in grade C. Ischemic HCC was 16/79 cases (20%) and plethoric HCC was 55/79 cases (70%). The median alpha-fetoprotein (AFP) (ng/mL) and des-gamma carboxy prothrombin (PIVKA-II) (AU/mL) value before SBRT for evaluable 73 patients with HCC were 12.7 (range; 0.8-8004) and 35 (range; 3.1-16900). The median indocyanine green retention rate at 15 min (ICG15) value before SBRT for evaluable 25 patients with HCC was 21.2% (range; 3–56.2%). This SBRT was the first treatment in 26/79 cases (33%) and was the first treatment about the same lesion as this SBRT in the additional 7 cases. About the primary tumor site of liver metastases, colo-rectum was 58.8%, lung was 9.8%, and stomach was 9.8%. The number of SBRT lesions was from 1 to 4 (solitary was 41/51 cases) for liver metastasis.

**Table 1:** Patient and tum or characteristics of SBRT

**Liver metastasis**	**N**	**%**	**HCC**		**N**	**%**
	51	100			79	100
Primary cancer			Stage			
Colon cancer	21	41.2		I	29	36.7
Rectal cancer	9	17.6		II	21	26.6
Lung cancer	5	9.8		III	5	6.3
Gastric cancer	5	9.8		IV	2	2.5
Cervical cancer	3	5.9		Recurrence	11	13.9
Breast cancer	3	5.9		NE	11	13.9
Pancreatic cancer	3	5.9				
Bile duct cancer	1	2.0				
Skin cancer	1	2.0				
Number of SRT				Chilid-Pugh before SBRT		
Single SRT	41	80.4		A	67	84.8
Two places	8	15.7		B	9	11.4
Tree	1	2.0		C	1	1.3
Four	1	2.0		NE	2	2.5
Sex						
Female	17	33.3			19	24
Male	34	66.7			60	75.9
Tumor diameter (mm)						
Range	13-54				6-70	
Median	26				27	
Performance status (ECOG)						
0	32	62.7			34	43.0
1	13	25.5			39	49.4
2	5	9.8			4	5.1
3	1	2.0			1	1.3
Age (years old)						
Range	33-90				38-95	
Median	73				73	
SRT total dose (Gy)						
Range	30-60				40-60	
Median	50				48	
BED-10 (Gy)						
Range	56-134.4				75-106	
Median	105.6				96.3	

*Abbreviation:NE* not evaluable.

### Treatment

**Figure 1:**
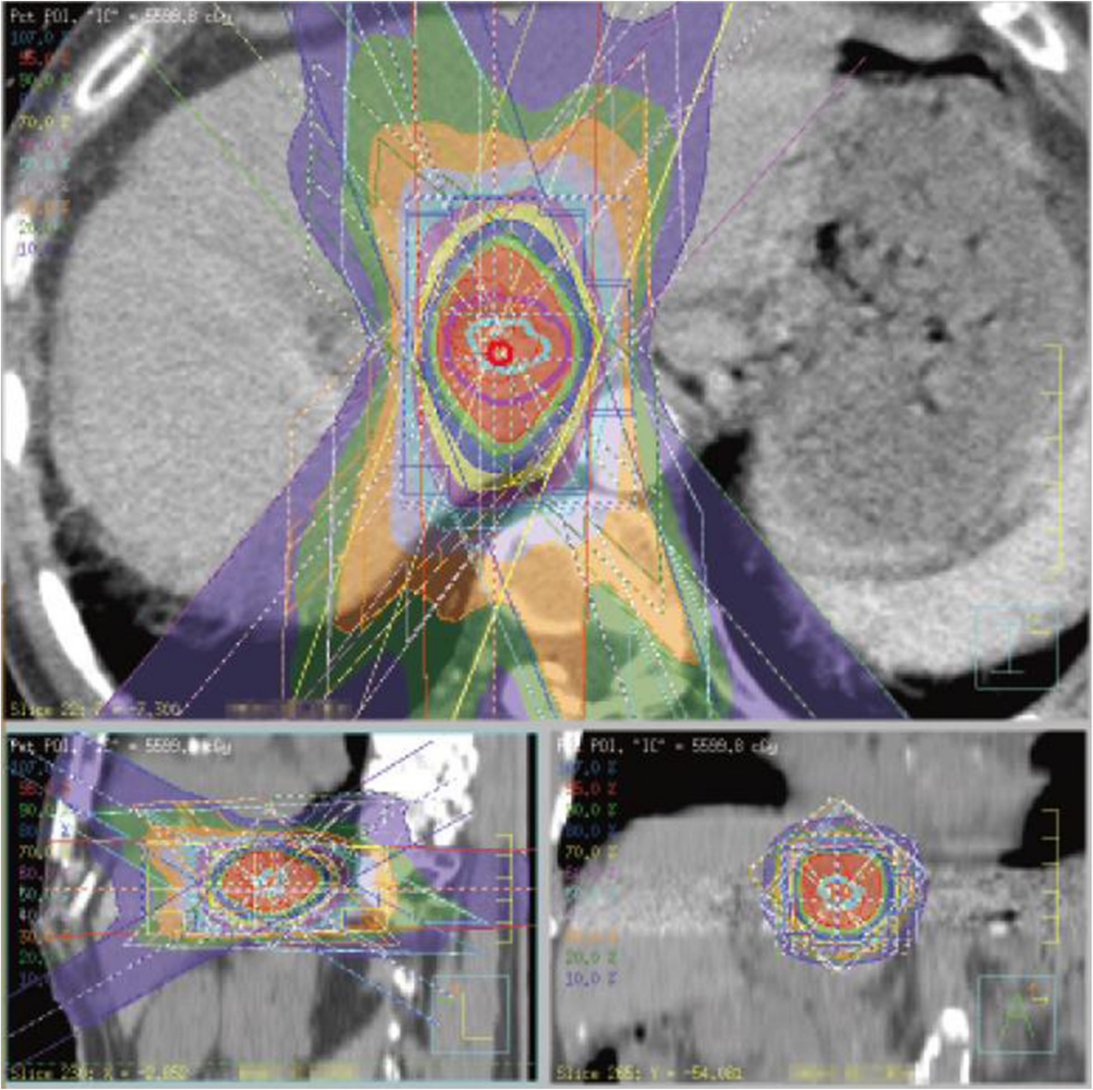
**Dose distribution of SBRT for liver tumor.** Sky blue line = ITV, purple line = PTV, red area = over 95% dose, green area = 90-95%, blue area = 80-90%, yellow area = 70-80%, purple area = 60-70%, sky blue area = 50-60%, orange area = 30-40%.

For treatment planning, abdominal pressure corsets such as body shell or vacuum cushion such as blue back were used, and it was confirmed that tumor motion was <1 cm. Then, the gross tumor volume (GTV) was delineated on the both inspiratory and expiratory planning CT images in the case of respiratory depression method. The breath-holding method was used in 36 cases, gating method in 10 cases, and respiratory depression method in 25 cases about HCC patients. The planning target volume (PTV) was configured considering respiratory movement, a set-up margin, and a sub-clinical margin (Figure 1). SBRT was performed with an X-ray beam linear accelerator of 6 MV. The total dose was delivered depending on judgment each institution. A collapsed cone (CC) convolution, superposition algorithm, or analytical anisotropic algorithm (AAA) was used for dose calculations.

The mode value of total irradiated dose was 48 Gy in 4 fractions (38/79 cases) (from 40 Gy in 4 fractions to 60 Gy in 10 fractions) for HCC and 48 Gy in 4 fractions (12/51 cases) and 52 Gy in 4 fractions (16/51 cases) (from 30 Gy in 3 fractions to 60 Gy in 8 fractions) for metastatic liver tumor. The biologically effective dose (BED) (α/β = 10 Gy) was 75–106 Gy (median: 96 Gy) for patients with HCC and 56–134 Gy (median: 106 Gy) with metastatic liver tumor (Table 1). The formula about BED_10_ was used; BED (Gy_10_) = nd (1 + d/α/β). In all 130 cases, CT registration like cone beam CT was performed each treatment.

SBRT was delivered using multiple non-coplanar static beams (using > 7 non-coplanar fields) generated by a linear accelerator or volumetric modulated arc therapy. Daily image guidance, by using either orthogonal X-rays or onboard CT imaging, was used to re-localize the target before treatment delivery.

Trans-catheter arterial chemoembolization (TACE) in 7 HCC patients, FOLFILI regimen (folinic acid, fluorouracil, plus irinotecan) in a metastatic liver tumor patient, or TAXOL® (paclitaxel) in a metastatic liver tumor patient was performed before SBRT. Oral TS-1 was combined concurrently with SBRT in an HCC patient.

### Follow up

Patients were seen monthly for 1 year after SBRT and tri-monthly thereafter. Laboratory tests were done at every visit. Treatment responses and intrahepatic recurrences were evaluated with dynamic contrast-enhanced CT or MRI every 3 months with modified Response Evaluation Criteria in Solid Tumors (mRECIST) [25]. Toxicity was evaluated with the Common Terminology Criteria for Adverse Events (CTCAE), version 4.0. Acute and sub-acute toxicities were defined as adverse events occurring within 3 months and 3–6 months, respectively, after SBRT. Late toxicities related with liver and other toxicities were defined as those occurring after 6–12 months and from 6 months to last follow-up, respectively. Laboratory tests included aspartate aminotransferase, total bilirubin, platelet count, and albumin.

Local recurrence was defined as progressive disease in mRECIST or the new appearance of a lesion within the PTV, and local control was defined as free of local recurrence. Local control was defined as freedom from local progression by mRECIST.

### Statistical analysis

Control and survival rates were calculated with Kaplan-Meier analysis. Log-rank testing was used to compare outcomes between the subsets of patients analyzed. Cox proportional hazards regression analysis was used for multivariate analysis. A *p*-value of <0.05 was considered significant. Data were analyzed with SPSS Statistics 20.0 (IBM Corp., Armonk, NY, USA). The points on survival curves by Kaplan Meier are a censored case.

## Results

### Eligible patients

The median follow-up time was 475.5 days (range; 101–2050 days) in patients with HCC and 212.5 days (range; 26–2713 days) with metastatic liver tumor. SBRT was performed as scheduled and was feasible in all patients. At the last follow-up, 48/79 cases (61%) were survival and 31/79 (39%) were dead for HCC and 42/51 cases (82%) were survival and 9/51 cases (18%) were dead for metastatic liver tumors.

### Treatment outcomes

Clinical results were shown in Table 2. As to the initial local effect, complete response (CR) and partial response (PR) were 45.6% and 35.4% in SBRT for HCC and 29.4% and 45.1% for metastatic liver tumor, respectively.

**Table 2:** Clinical results of SBRT

	**N**	**%**	**N**	**%**
	**Liver metastasis**		**HCC**	
First local effect				
CR	15	29.4	36	45.6
PR	23	45.1	28	35.4
MR	2	3.9	0	0
NC	6	11.8	9	11.4
PD	0	0	4	5.1
NE	5	9.8	2	2.5
Local progress				
With	10	19.6	14	17.7
Without	37	72.5	63	79.7
NE	4	7.8	2	2.5

*Abbreviation:CR* complete response, *PR* partial response, *MR* minor response, *NC* no change, *PD* progress disease, *NE* not evaluable.

**Figure 2:**
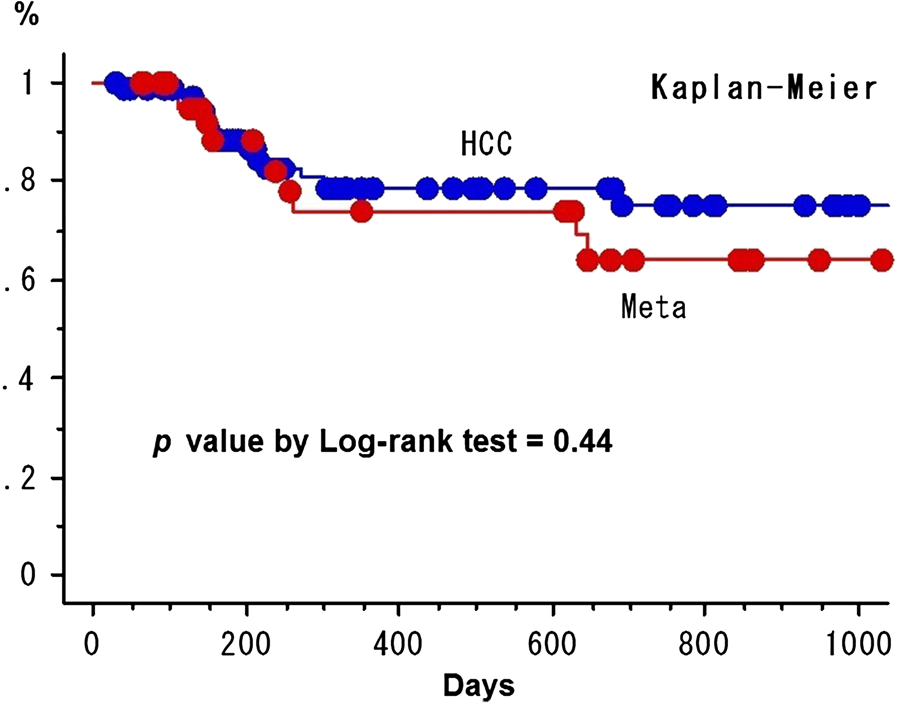
**Local control curves between SBRT for hepatic cell carcinoma and metastatic liver tumor.** The points on survival curves are a censored case.

**Figure 3:**
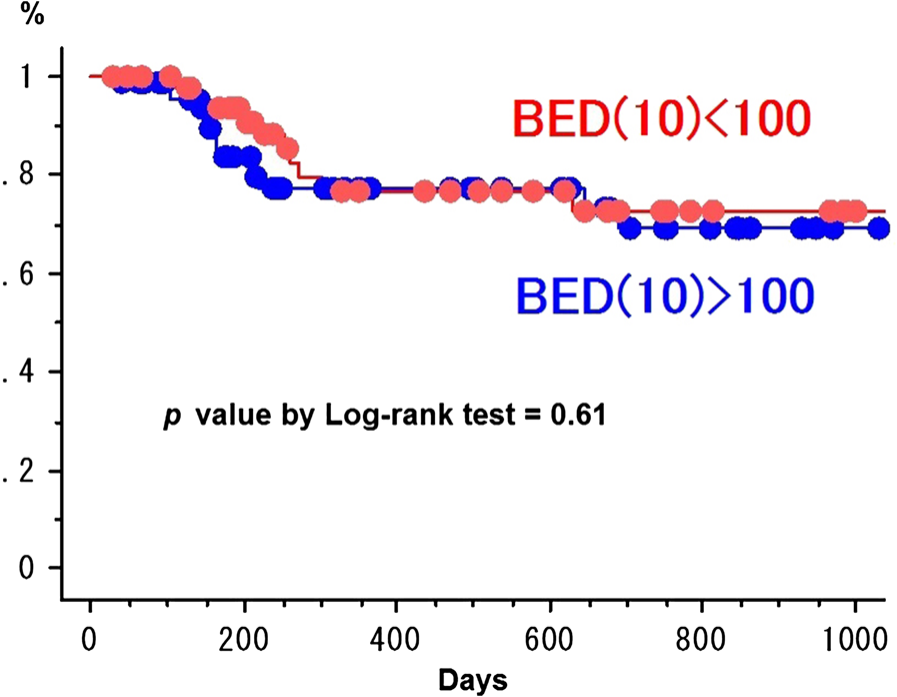
**Local control curves between BED (10) > 100 Gy and < 100 Gy.** The points on survival curves are a censored case.

**Figure 4:**
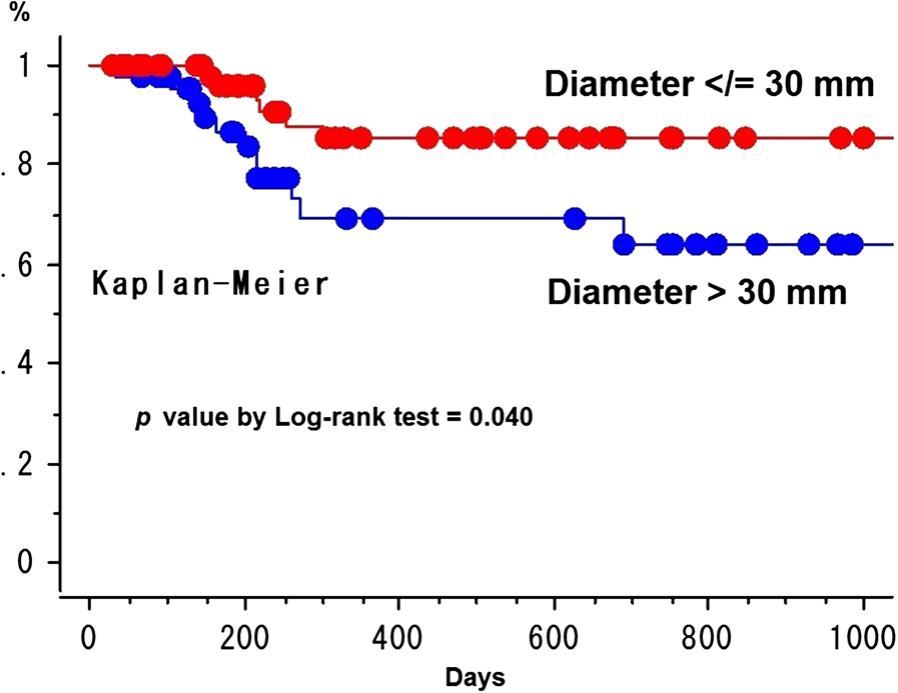
**Local control curves between maximum tumor diameter > 30 mm and </=30 mm.** The points on survival curves are a censored case.

The 2-year cumulative LCR for HCC and metastatic liver tumor was 74.8% ± 6.3% (standard error) and 64.2 ± 9.5% (*p* = 0.44) (Figure 2). The LCR was not different between BED_10_ ≥ 100 Gy (69.0% ± 7.6% at 2 years) vs. < 100 Gy (72.4% ± 7.7%) in all 130 patients (*p* = 0.61) (Figure 3). The LCR was not different between HCC (68.2% ± 11.2%) vs. liver metastasis (68.3% ± 11.2%) in 70 patients with the higher BED_10_ ≥ 100 Gy (*p* = 0.96). The LCR was not different between BED_10_ ≥ 100 Gy (68.3% ± 11.2%) vs. < 100 Gy (46.5% ± 16.9%) in 51 patients with liver metastasis (68.2% ± 11.2% vs. 79.2% ± 7.7%, *p* = 0.72) and in 79 patients with HCC (*p* = 0.43). In all 130 patients, the LCR was not different between maximum tumor diameter > 20 mm vs. ≤ 20 mm (70.6% ± 7.6% vs. 83.5% ± 7.6%, *p* = 0.28) and ≥ 40 mm vs. < 40 mm (55.4% ± 17.2% vs. 79.8% ± 5.1%, *p* = 0.32) except for > 30 mm vs. ≤ 30 mm (64.1% ± 9.1% vs. 85.2% ± 5.6%, *p* = 0.040) (Figure 4). The LCR was not different between BED_10_ ≥ 100 Gy (66.2% ± 33.8%) vs. < 100 Gy (62.3% ± 12.6%) in 41 patients with the bigger tumor diameter > 30 mm (*p* = 0.78). The LCR was not different between older (>70 y.o.) vs. younger (≤70 y.o.) (74.4% ± 6.2% vs. 70.6% ± 8.9%, *p* = 0.76).

By multivariate analysis (Cox proportional hazards regression analysis), the maximum tumor diameter > 30 mm vs. ≤ 30 mm (other covariates were BED_10_ ≥ 100 Gy vs. <100 Gy of *p* = 0.70, age >70 y.o. vs. ≤ 70 y.o. of *p* = 0.73, HCC vs. metastatic liver tumor of *p* = 0.52) was the only significant factor for LCR (*p* = 0.047, 95% CI = 1.014-7.546).

**Figure 5:**
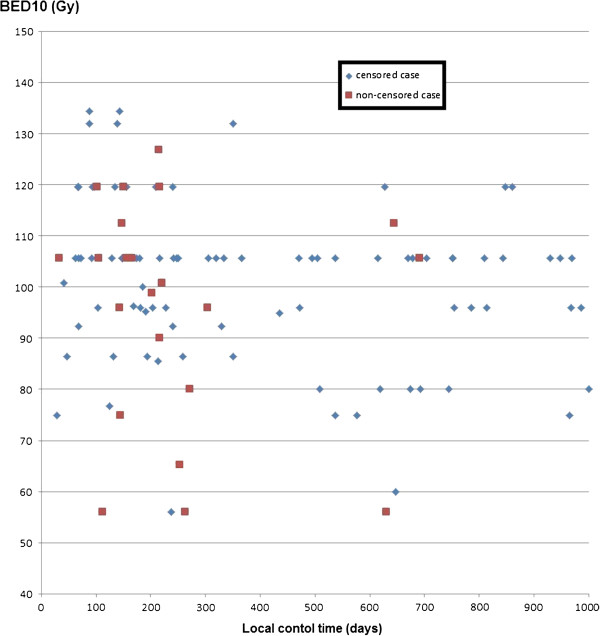
Scatter diagram between BED10 (Gy) and local control time (days).

The scatter diagram between BED_10_ and local control time was shown in Figure 5. There was no correlation between BED_10_ and local control time. We didn’t show the fact that the higher BED_10_ was, the longer local control time was.

**Figure 6:**
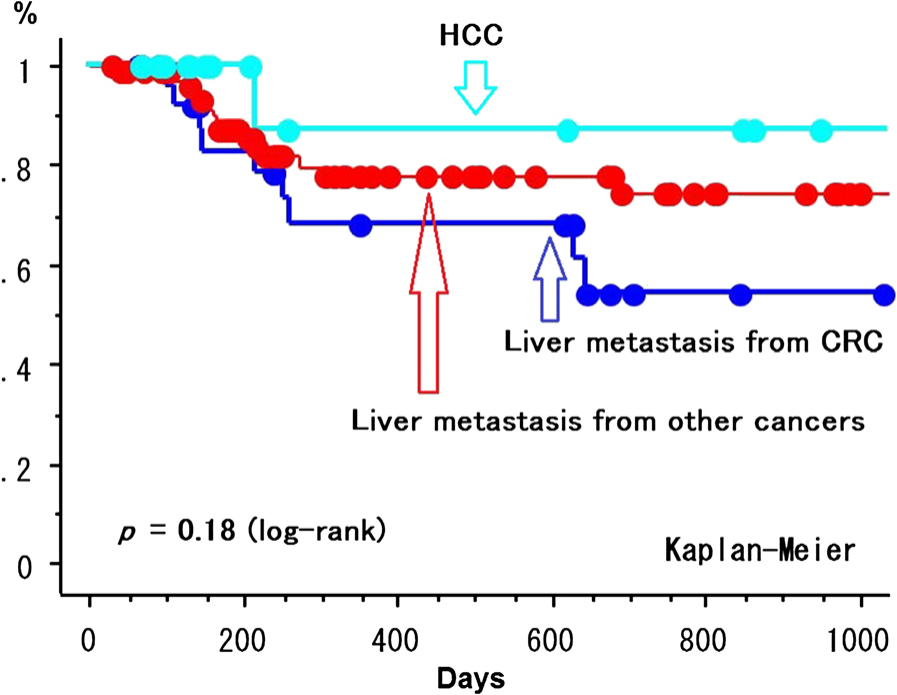
Local control curves among HCC, liver metastases from colorectal cancer, and from other cancers.

The 2-year overall survival (OS), cause specific survival (CSS), disease free survival (DFS), and distant metastatic free survival (DMF) were 52.9% ± 7.1%, 69.0% ± 6.9%, 39.9% ± 6.9%, and 76.3% ± 6.6% in 79 patients with HCC, respectively (Figure 6). The number of patients at risk was 43, 21, 9, and 3 at 1-, 2-, 3-, and 4-year in OS, respectively. The 2-year OS was 71.9% ± 9.4% in 51 patients with metastatic liver tumor.

The 2-year cumulative LCR for HCC (n = 79) vs. metastatic liver tumor from colorectal cancer (n = 30) vs. from other cancers (n = 21) was 74.1% ± 6.2% vs. 54.2% ± 11.8% vs. 87.5% ± 11.7% (*p* = 0.18 by comparison among three groups, *p* = 0.12 between colorectal and other cancers, and *p* = 0.16 between HCC and colorectal cancer).

### Treatment-related toxicity

All SBRT were completed without toxicity during RT period. There was no Grade 5 toxicity. Nine patients (7%) experienced Grade 2–4 gastrointestinal toxicity. Three patients had Grade 2 gastric inflammations at both 1 Mo (40 Gy in 4 fractions and 60 Gy in 10 fractions) and one gastric ulcer at 27 Mo (60 Gy in 10 fractions). Four had Grade 3 intestinal tract bleedings at 5 Mo (50 Gy in 5 fractions) and 6 Mo (40 Gy in 4 fractions) and transverse colon ulceration at 5 Mo (60 Gy in 10 fractions) and duodenal ulcer at 17 Mo (48 Gy in 4 fractions) without chemotherapy in all 4 cases. One patient had Grade 4 gastro-duodenal artery rupture at 6 Mo after SBRT of 48 Gy in 4 fractions without chemotherapy. One patient complained of chest wall pain after SBRT of 45.2 Gy in 4 fractions combined with TACE.

No significant (≥ grade 3) liver enzyme elevation was observed during treatment. No classic RILD was observed.

## Discussion

This is a retrospective study to review 130 patients with primary or metastatic liver cancers treated at 20 institutions extracted from the database of JRS-SBRTSG. The primary aim of the paper is to report outcome in terms of survival, local control, and toxicity. Overall survivals in this study of 53% for HCC (n = 79) and 72% for liver metastases (n = 51) at 2 year after SBRT were almost satisfactory (median follow-up was 16 months), but there were various biases in that the candidates included frail patients contraindicated due to decompensated cirrhosis and older patients with a median age of 73 years. It was the reason why only LCR was performed for the factor analysis in this study.

The local controls after stereotactic body radiotherapy for liver tumor were 65% to 100% in HCC and 56% to 100% in metastatic liver tumor. Results of phase I/II studies and retrospective series of SBRT for HCC patients indicated high local control rates of 90-100% [26-29]. In this study, local recurrence was seen at within 8 months in almost all cases and at 20 to 23 months in some cases. The LCR of HCC in this study was slightly poor and could hardly have been more different from that of metastatic liver tumor. We showed the summary of LC after SBRT for liver tumor in Table 3.

**Table 3:** Summary of local control after stereotactic body radiotherapy for liver tumor

**First author**	**Ref.**	**Target**	**Year**	**Case no.**	**Total RT dose**	**Fr no.**	**LC**	**Timing of LC**
Blomgren H	[30]	HCC	1995	20	15-45 Gy	1-5	80%	1.5-38 mo
Tse RV	[26]	HCC & IHC	2008	41	36 Gy	6	65%	12 mo
Cardenes HR	[31]	HCC	2010	17	36-48 Gy	3	100%	10-42 mo
Kwon JH	[32]	HCC	2010	42	30-39 Gy	3	68%	36 mo
Louis C	[33]	HCC	2010	25	45 Gy	3	95%	24 mo
Seo YS	[34]	HCC	2010	38	33-57 Gy	3-4	66%	24 mo
Andolino DL	[28]	HCC	2011	60	40 Gy, 44 Gy	5	90%	24 mo
						3		
Kang JK	[29]	HCC	2012	50	42-60 Gy	3	95%	24 mo
Takeda A	[21]	HCC	2013	63	35-40 Gy	5	92%	36 mo
Herfarth KK	[10]	ML	2001	37	14-26 Gy	*NA*	78%	5.7 mo
Wada H	[11]	ML	2004	34	45 Gy	3	86%	12 mo
Kavanagh BD	[12]	ML	2006	36	60 Gy	3	93%	18 mo
Hoyer M	[13]	ML	2006	64	45 Gy	3	63%	24 mo
Katz AW	[14]	ML	2007	69	30-55 Gy	*NA*	57%	20 mo
Lee MT	[16]	ML	2009	68	27.7-60 Gy	6	71%	12 mo
Rusthoven KE	[17]	ML	2009	47	36-60 Gy	3	92%	24 mo
Rule W	[18]	ML	2011	27	30 Gy,	3	56%	24 mo
					50 Gy,	5	89%	
					60 Gy	5	100%	
Chang DT	[19]	ML	2011	65	46-52 Gy	3	90%	12 mo
Fumagalli I	[20]	ML	2012	90	15 Gy	3	66%	24 mo

*Abberiviation:HCC* hepatocellular carcinoma, *IHC* intrahepatic cholangiocarcinoma, *ML* metastatic liver tumor, *RT* radiotherapy, Fr = fractions, LC = local control, *mo* months.

LCR might be overestimated using cumulative LCR like the present report because patients who died without the evidence of local recurrence were excluded. Since the pure LCR want to be calculated, the patients who died without local recurrence were treated as a censored case. Takeda *et al.*[21] reported that LCR after SBRT for lung metastases from colorectal cancer with a 2-year LCR of 72% was worse than that for primary lung cancer and also in the present study, LCR for liver metastases from colorectal cancer was slightly worse than that for HCC or liver metastases from other cancers, although there was no significant difference. The patient number at this time may be too small to detect the significant differences on LCR among three groups.

To improve our results of local control and so on, we may increase radiation dose. The median BED_10_ in this study was 96 Gy for patients with HCC and 106 Gy with metastatic liver tumor. Although it is natural that BED_10_ is over 100 Gy in the SBRT for lung tumor, the fact may be not true of the SBRT for liver tumor. Although the aim of SBRT is to deliver a high ablative dose to destroy tumor cells, the optimal treatment dose should be determined based on both tumor control and long-term safety because radiation damage to the normal liver tissue is dose-volume-dependent [35,36]. In SBRT for liver tumors, the prescribed dose and fraction vary across studies, ranging from 24–60 Gy in 2–6 fractions, and most studies focused predominantly on liver metastases [37]. Since metastatic lung tumors require dose escalation due to relatively low radio-sensitivity [38], increasing the dose to metastatic liver tumors appears to be reasonable, and patients with normal liver function treated with SBRT have rarely developed RILD. In contrast, dose escalation in HCC patients with decompensated cirrhotic liver disease may be disadvantageous with respect to normal liver tolerance. A dose-control relationship has been described for patients treated with SBRT for liver and lung metastases. In an analysis of 246 lesions treated with three-fraction SBRT for primary or metastatic tumors within the lung or liver, McCammon *et al.*[39] demonstrated significant improvement in local control with increasing dose and the 3-year local control rate in their series was 89.3% for those lesions that received 54 to 60 Gy versus 59% and 8.1% for lesions that received 36 to 53.9 Gy and less than 36 Gy, respectively (*p* < 0.01). Tekeda *et al.*[40] used 35–40 Gy in 5 fractions based on baseline liver function and liver volume receiving ≥20 Gy of SBRT for untreated solitary HCC patients.

By multivariate analysis, the maximum tumor diameter > 30 mm vs. ≤ 30 mm was only one prognostic factor for LCR. According to Rusthoven *et al.*[17], actuarial in-field local control rates at one & two years after SBRT of 60 Gy in 3 fractions for the treatment of 47 patients with one to three hepatic metastases (63 lesions) were 95% & 92% and 2-year local control was 100% among lesions with maximal diameter of 3 cm or less.

However, this study has some limitations in that it is a retrospective and multi-institutional series with a relatively short follow-up period. The group is very heterogeneous including primary and metastatic liver tumors. That is why the irradiated dose and the follow-up method are inconsistent, too. The reason why there was no difference by the stratification of irradiated dose may be that in this study the problem of algorithm or prescription point can be integrated. We are planning to start a multi-institutional prospective large-scale clinical trial that standardized these factors.

## Conclusions

There was no difference in LCR between liver metastasis vs. HCC and the higher vs. lower BED_10_ against SBRT for liver cancer except for the bigger vs. smaller tumor diameter. SBRT is a safe treatment and may be an alternative option for patients with liver tumor unfit for resection or RFA. Further prospective studies are warranted to validate the effect of SBRT for liver tumor.

## Competing interests

The authors have no conflict of interest to disclose with respect to this presentation.

## Authors’ contributions

HY and HO carried out the molecular genetic studies, participated in the sequence alignment and drafted the manuscript. YM, NM, YM, TN, and TK were gave clinical data in their own institution and corrected the manuscript. KN corrected the manuscript. All authors read and approved the final manuscript.
